# Obituary

**Published:** 1919-11

**Authors:** 


### OBITUARY

---

NEWELL SILL JENKINS, D.D.S. Died September 25, 1919, at Havre, France, in his seventy-ninth year, after a very brief illness. Dr. Jenkins was a graduate of the Baltimore College of Dental Surgery, class of 1863. He began practice in Bangor, Maine, soon after graduating but moved to Germany after two or three years and settled in Dresden, where he established one of the great American dental practices. Because of his skill and remarkable social qualities he attracted the choicest clientele in Europe.

He sold his practice, after nearly forty years, to an American dentist, for the largest sum ever paid for a dental practice. Dr. Jenkins was one of the men who did so much to introduce American standards of dentistry in Europe. He contributed not only to the social position of American dentists but materially to the technical art of his profession. He was a great admirer of Dr. W. D. Miller, also an intimate personal friend. He was a most lovable man and his charming and courteous attitude toward everyone was of such a character as to make him probably the best known and most highly respected American dentist in Europe. He added many things of value to the art and science of dentistry. His wife, two daughters and his son, L. A. Jenkins, of New Haven, survive him.

